# The incidence and characteristics of accelerated knee osteoarthritis among women: the Chingford cohort

**DOI:** 10.1186/s12891-020-3073-3

**Published:** 2020-01-31

**Authors:** Jeffrey B. Driban, Raveendhara R. Bannuru, Charles B. Eaton, Tim D. Spector, Deborah J. Hart, Timothy E. McAlindon, Bing Lu, Grace H. Lo, Nigel K. Arden

**Affiliations:** 1 Division of Rheumatology, Allergy & Immunology, Tufts Medical Center, 800 Washington Street, Box #406, Boston, MA 02111 USA; 2 Center for Primary Care and Prevention, Alpert Medical School of Brown University, 111 Brewster St, Pawtucket, RI 02860 USA; 3 Department of Twin Research and Genetic Epidemiology, King’s College London, St Thomas’ Campus, Lambeth Palace Road, London, SE1 7EH UK; 4 Division of Rheumatology, Immunology and Allergy, Brigham and Women’s Hospital and Harvard Medical School, 75 Francis Street, Boston, MA 02115 USA; 5 Medical Care Line and Research Care Line, Houston Health Services Research and Development (HSR&D) Center of Excellence Michael E. DeBakey VAMC, 2002 Holcombe Blvd, Houston, TX 77030 USA; 6 Section of Immunology, Allergy, and Rheumatology, Baylor College of Medicine, 1 Baylor Plaza, BCM-285, Houston, TX 77030 USA; 7 Nuffield Department of Orthopaedics, Rheumatology & Musculoskeletal Sciences, University of Oxford, Windmill Road, Headington, Oxford, OX3 7HE UK

**Keywords:** Phenotype, Epidemiology, Total knee replacement

## Abstract

**Background:**

Prior research on accelerated knee osteoarthritis (AKOA) was primarily confined to the Osteoarthritis Initiative, which was enriched with people with risk factors for knee osteoarthritis (KOA). It is unclear how often AKOA develops in a community-based cohort and whether we can replicate prior findings from the Osteoarthritis Initiative in another cohort. Hence, we determined the incidence and characteristics of AKOA among women in the Chingford Study, which is a prospective community-based cohort.

**Methods:**

The Chingford Study had 1003 women with quinquennial knee radiographs over 15 years. We divided the 15-year observation period into three consecutive 5-year phases. Within each 5-year phase, we selected 3 groups of participants among women who started a phase without KOA (Kellgren-Lawrence [KL] < 2): 1) incident AKOA developed KL grade ≥ 3, 2) typical KOA increased radiographic scoring (excluding AKOA), and 3) no KOA had the same KL grade over time. Study staff recorded each participant’s age, body mass index (BMI), and blood pressure at baseline, 5-year, and 10-year study visits. We used multinomial logistic regression models to test the association between groups (outcome) and age, BMI, and blood pressure at the start of each phase. The cumulative incidences and odds ratios (OR) from each phase were pooled using a fixed-effect meta-analysis model.

**Results:**

The person-based cumulative incidence of AKOA was 3.9% over 5 years (pooled estimate across the three 5-year phases). Among incident cases of KOA, AKOA represented ~ 15% of women with incident KOA. Women with AKOA were older than those with typical (OR = 1.56, 95%CI = 1.16–2.11) or no KOA (OR = 1.84, 95%CI = 1.40–2.43). Women with AKOA had a greater BMI than those without KOA (OR = 1.52, 95%CI = 1.17–1.97). We observed no association between group and blood pressure.

**Conclusions:**

In a community-based cohort, > 1 in 7 women with incident KOA had AKOA. Like the Osteoarthritis Initiative, people with AKOA were more likely to have greater age and BMI.

## Background

While knee osteoarthritis (KOA) is perceived as a slowly progressive disorder, we demonstrated within the Osteoarthritis Initiative (OAI) that 1 in 5 cases of incident KOA experience an accelerated onset and progression from no radiographic disease to advanced-stage disease (definite joint space narrowing and osteophyte) within 4 years [1]. Adults with accelerated KOA (AKOA) have greater pain and disability compared to adults with typical KOA – starting years prior to radiographic disease onset [2, 3]. More than 1 in 14 adults with AKOA receive a knee replacement within 2.5 years after the first evidence of radiographic progression [4].

The OAI’s annual clinical visits, which included imaging, provided an exceptional opportunity to characterize AKOA but it remains unknown if this subset is unique to the OAI, which is a cohort enriched with risk factors for KOA. Our overall goal was to determine if AKOA is present in a community-based cohort and whether prior findings about AKOA from the OAI can be replicated in another cohort. Hence, we wanted to determine the incidence of AKOA among women in a prospective community-based cohort. Secondly, we sought to determine if age, body mass index (BMI), and blood pressure were associated with incident AKOA. Finally, we sought to report the frequency of knee replacements among women with and without AKOA. We hypothesized that women with AKOA would be older and have a greater BMI than peers with typical or no onset of KOA. We also hypothesized that blood pressure would be associated with AKOA based on an observed trend in the OAI, which failed to reach statistical significance [1].

## Methods

### Study sample

We assessed 1003 women in the Chingford Study [5], which obtained quinquennial knee radiographs over 15 years. In brief, the Chingford Study was started in Chingford, North London, United Kingdom by contacting all women 45 to 64 years of age from a register of a large general practice in 1988 to 1989. The Chingford Study has met all criteria for ethical standards regarding human studies as described in the 1964 Declaration of Helsinki and all amendments. The Outer North East London Research Ethics Committee approved the study. Each study participant provided written informed consent before participating.

### Definition of incident accelerated and typical knee osteoarthritis

We divided the 15-year observation period into three consecutive 5-year phases. Within each 5-year phase, we selected 3 groups of participants among women who started with a knee without definite radiographic signs of KOA (Kellgren-Lawrence [KL] < 2): 1) incident AKOA developed KL grade ≥ 3 (definite osteophyte and joint space loss) within 5 years [6], 2) typical KOA increased radiographic scoring within 5 years (i.e., KL = 0 to 1, 0 to 2, 1 to 2), and 3) no KOA had the same KL grade over 5 years. We selected a 5-year phase based on the available images in the Chingford Study and our preliminary analysis of OAI data, which indicated that adding an extra year to our previously validated definition of AKOA over 4 years would only yield seven new cases of AKOA (4% increase from 193 knees to 200 knees in the OAI). For person-based analyses, we required both knees to have no radiographic KOA (KL = 0 or 1) at the start of a phase and classified women based on whether they had a knee develop AKOA, typical KOA (but not AKOA), or no KOA in both knees.

### Knee radiographs

Radiographic disease severity was based on weight-bearing anteroposterior knee radiographs. A detailed description of the KL grading system has been reported for the Chingford Study (e.g., KL grade = 3 represented the presence of joint space loss and osteophytes) [7]. Inter-observer agreement (kappas) were 0.56 to 0.80 [7].

### Clinical measures

We selected risk factors and an outcome that were assessed in the OAI and consistently collected over time in the Chingford Study. Staff collected at each visit a participant’s weight, height, and blood pressure. Participants self-reported total knee replacements on annual follow-up questionnaires.

### Statistical analyses

We calculated person-based and knee-based cumulative incidence of AKOA over each 5-year phase and the percentage of incident KOA that was AKOA. We also describe the incidence of total knee replacements by group during each phase. All subsequent analyses were person-based. We used multinomial logistic regression models to test the person-based association between groups (outcome) and 4 risk factors at the start of each phase: age, BMI, and systolic and diastolic blood pressure (unadjusted and adjusted for the other 3 risk factors). We calculated odds ratio and 95% confidence interval for each variable per one standard deviation using SAS Enterprise 7.15 (Cary, NC, USA). Cumulative incidences and odds ratios from each period were pooled using fixed-effect meta-analysis models to estimate the cumulative incidence and odds ratios. We also performed a sensitive analysis with random-effect meta-analysis models.

## Results

Overall, the Chingford Study started with a mean (standard deviation) age of 53 (6) years, BMI of 25.0 (3.6) kg/m^2^, systolic blood pressure of 123 (20) mmHg, and diastolic blood pressure of 75 (10) mmHg. The person-based cumulative incidence of AKOA (pooled estimate) across the three 5-year phases was 3.9% (Table 1 and 2). Among incident cases of KOA, AKOA represented ~ 15% of all people with incident KOA and ~ 17% of knees with incident KOA. During the 10 years after the first phase, 5 out of 24 (21%) women with AKOA received a total knee replacement compared with 2 out 102 (2%) women with typical KOA and 8 out of 966 women with no KOA (0.9%). During the 5 years after the second phase, 1 out of 27 (4%) women with AKOA received a total knee replacement compared with 1 out of 215 (0.5%) women with typical KOA and 3 out of 685 (0.4%) with no KOA.

**Table 1:** Cumulative Incidence of Accelerated and Typical Knee Osteoarthritis (KOA) over 5-year intervals

	Accelerated KOA	Typical KOA	No KOA
Person-based			
Phase 1: Year 1 - Year 5 (*n* = 715)	25 (3.5%)	93 (13.0%)	597 (83.5%)
Phase 2: Year 5 - Year 10 (*n* = 574)	18 (3.1%)	169 (29.4%)	387 (67.4%)
Phase 3: Year 10 - Year 15 (*n* = 377)	20 (5.3%)	83 (22.0%)	274 (72.7%)
Pooled Estimate (95% CI)	3.9% (3.0 to 4.9)	21.7% (19.7 to 23.8)	
Knee-based			
Phase 1: Year 1 - Year 5 (*n* = 1508)	38 (2.5%)	159 (10.5%)	1311 (86.9%)
Phase 2: Year 5 - Year 10 (*n* = 1255)	40 (3.2%)	301 (24.0%)	914 (72.8%)
Phase 3: Year 10 - Year 15 (*n* = 867)	48 (5.5%)	146 (16.8%)	673 (77.6%)
Pooled Estimate (95% CI)	3.7% (3.1 to 4.3)	17.6% (16.3 to 18.9)

**Table 2:** Frequency of Unilateral versus Bilateral Outcomes Among People with or without Accelerated, Typical or No Knee Osteoarthritis (KOA)

Person-based Outcome	Laterality of Outcome	n (%)
Phase 1: Year 1- Year 5 (*n* = 715)		
Accelerated KOA (*n* = 25)	Unilateral (contralateral = Typical KOA)	9 (36%)
	Unilateral (contralateral = No KOA)	9 (36%)
	Bilateral	7 (28%)
Typical KOA (*n* = 93)	Unilateral	64 (69%)
	Bilateral	29 (31%)
No KOA (*n* = 597)	Unilateral	0 (0%)
	Bilateral	597 (100%)
Phase 2: Year 5- Year 10 (*n* = 574)		
Accelerated KOA (*n* = 18)	Unilateral (contralateral = Typical KOA)	5 (28%)
	Unilateral (contralateral = No KOA)	4 (22%)
	Bilateral	9 (50%)
Typical KOA (*n* = 169)	Unilateral	83 (49%)
	Bilateral	86 (51%)
No KOA (*n* = 387)	Unilateral	0 (0%)
	Bilateral	387 (100%)
Phase 3: Year 10 - Year 15 (*n* = 377)		
Accelerated KOA (*n* = 20)	Unilateral (contralateral = Typical KOA)	3 (15%)
	Unilateral (contralateral = No KOA)	11 (55%)
	Bilateral	6 (30%)
Typical KOA (*n* = 83)	Unilateral	51 (61%)
	Bilateral	32 (39%)
No KOA (*n* = 274)	Unilateral	0 (0%)
	Bilateral	274 (100%)

Reported percentage is based on the number within each row divided by the number of people with that outcome (i.e., AKOA, Typical KOA, or No KOA)

Across the 3 phases we found that women with AKOA were older than those with typical (OR = 1.56 per one standard deviation of age) or no KOA (OR = 1.84). Furthermore, women with AKOA had a greater BMI than those with no KOA (OR = 1.52 per one standard deviation of BMI; Table 3). The sensitivity analyses with random effects were consistent with the results in Tables 1 and 3 (Additional file 1: Tables S1 and S2).

**Table 3:** Baseline Person-Based Characteristics Associated with Accelerated Knee Osteoarthritis (AKOA) During Each Phase

Phase 1: Year 1 - Year 5 (*n* = 715; 702 in model)							
Variable	No KOA (*n* = 585)	Typical KOA (*n* = 92)	AKOA (*n* = 25)	AKOA vs No KOA	AKOA vs Typical KOA	AKOA vs No KOA	AKOA vs Typical KOA
	m (sd)	m (sd)	m (sd)	OR (95% CI)	OR (95% CI)	aOR (95% CI)	aOR (95% CI)
Age (sd = 5.8 years)	53.7 (5.9)	55.6 (5.3)	56.7 (5.0)	**1.65 (1.10, 2.47)**	1.20 (0.77, 1.87)	**1.88 (1.21, 2.93)**	1.29 (0.79, 2.10)
BMI (sd = 3.9 kg/m^2^)	24.9 (3.7)	26.4 (4.6)	25.9 (3.8)	1.30 (0.90, 1.87)	0.91 (0.61, 1.34)	1.30 (0.87, 1.93)	0.87 (0.57, 1.34)
Systolic BP (sd = 20 mmHg)	127 (21)	128 (19)	126 (15)	0.95 (0.63, 1.44)	0.90 (0.58, 1.42)	**0.44 (0.21, 0.91)**	0.53 (0.24, 1.16)
Diastolic BP (sd = 11 mmHg)	78 (11)	79 (11)	80 (8)	1.22 (0.84, 1.77)	1.13 (0.74, 1.71)	1.75 (0.91, 3.36)	1.78 (0.87, 3.62)
Phase 2: Year 5 - Year 10 (n = 574; 539 used in model)							
Variable	No KOA (*n* = 365)	Typical KOA (*n* = 157)	AKOA (*n* = 17)	AKOA vs No KOA	AKOA vs Typical KOA	AKOA vs No KOA	AKOA vs Typical KOA
	m (sd)	m (sd)	m (sd)	OR (95% CI)	OR (95% CI)	aOR (95% CI)	aOR (95% CI)
Age (sd = 5.8 years)	57.2 (5.6)	58.1 (5.8)	62.1 (6.5)	**2.33 (1.39, 3.90)**	**1.98 (1.17, 3.35)**	**2.87 (1.61, 5.11)**	**2.54 (1.41, 4.56)**
BMI (sd = 4.1 kg/m^2^)	25.5 (3.8)	26.4 (4.6)	29.1 (4.6)	**1.96 (1.35, 2.86)**	**1.55 (1.06, 2.26)**	**2.76 (1.69, 4.52)**	**2.26 (1.37, 3.71)**
Systolic BP (sd = 19 mmHg)	123 (19)	127 (20)	125 (16)	1.09 (0.67, 1.77)	0.89 (0.54, 1.46)	0.89 (0.47, 1.68)	0.81 (0.42, 1.54)
Diastolic BP (sd = 12 mmHg)	74 (12)	75.6 (13)	69.4 (15)	0.66 (0.38, 1.14)	**0.56 (0.32, 0.99)**	**0.39 (0.20, 0.75)**	**0.38 (0.20, 0.74)**
Phase 3: Year 10 - Year 15 (*n* = 377; 374 used in model)							
Variable	No KOA (*n* = 272)	Typical KOA (*n* = 82)	AKOA (*n* = 20)	AKOA vs No KOA	AKOA vs Typical KOA	AKOA vs No KOA	AKOA vs Typical KOA
	m (sd)	m (sd)	m (sd)	OR (95% CI)	OR (95% CI)	aOR (95% CI)	aOR (95% CI)
Age (sd = 5.5 years)	61.4 (5.5)	61.1 (5.1)	63.9 (6.4)	1.52 (0.98, 2.35)	**1.62 (1.01, 2.60)**	1.38 (0.88, 2.15)	1.35 (0.83, 2.22)
BMI (sd = 3.9 kg/m^2^)	25.5 (3.7)	26.7 (4.4)	26.1 (3.8)	1.17 (0.75, 1.83)	0.87 (0.54, 1.40)	1.08 (0.67, 1.74)	0.78 (0.47, 1.31)
Systolic BP (sd = 21 mmHg)	135 (23)	131 (15)	146 (24)	**1.47 (1.02, 2.12)**	**1.87 (1.22, 2.89)**	1.32 (0.78, 2.27)	**2.13 (1.14, 4.01)**
Diastolic BP (sd = 11 mmHg)	80 (11)	81 (10)	84 (13)	1.36 (0.89, 2.10)	1.33 (0.83, 2.11)	1.05 (0.57, 1.91)	0.82 (0.43, 1.58)
Meta-Analysis of Baseline Characteristics from the 3 Phases							
Variable				AKOA vs No KOA	AKOA vs Typical KOA	AKOA vs No KOA	AKOA vs Typical KOA
				OR (95% CI)	OR (95% CI)	aOR (95% CI)	aOR (95% CI)
Age (sd = 5.8 years)				**1.75 (1.35 to 2.26)**	**1.52 (1.16 to 2.01)**	**1.84 (1.40 to 2.43)**	**1.56 (1.16 to 2.11)**
BMI (sd = 3.9 kg/m^2^)				**1.47 (1.17 to 1.84)**	1.11 (0.87 to 1.40)	**1.52 (1.17 to 1.97)**	1.13 (0.86 to 1.48)
Systolic BP (sd = 20 mmHg)				1.18 (0.93 to 1.50)	1.18 (0.91 to 1.53)	0.90 (0.63 to 1.28)	1.06 (0.72 to 1.57)
Diastolic BP (sd = 11 mmHg)				1.11 (0.87 to 1.43)	1.01 (0.77 to 1.33)	0.91 (0.63 to 1.31)	0.79 (0.53 to 1.16)

*KOA* knee osteoarthritis, *m* mean, *sd* standard deviation, *OR* odds ratio (per 1 standard deviation), *95% CI* 95% confidence interval, *aOR* adjusted odds ratio (adjusted for other variables in table), *BMI* body mass index, *BP* blood pressure

All boldface entries are statistically significant (*p* < 0.05) with confidence intervals that don't include 1.00

## Discussion

Most of the prior research on AKOA was confined to the OAI, which was enriched with people with symptomatic KOA or risk factors for KOA. This was our first endeavor to explore if AKOA is present in a community-based cohort. Furthermore, we sought to confirm our prior findings regarding the incidence of AKOA and its relationship with key risk factors (i.e., age, BMI, blood pressure) [1, 8, 9] and outcomes (total knee replacement) [4]. We found that AKOA represented more than 1 in 7 women with incident KOA. Furthermore, women with AKOA were more likely to have greater age and BMI prior to disease onset and perhaps more likely to receive a subsequent knee replacement. These findings offer the first estimates of the incidence of AKOA among a community-based cohort and confirm associations previously detected among OAI participants.

The pooled estimate of cumulative incidence of AKOA over 5 years was 3.9% in Chingford, which was comparable to the cumulative incidence from the OAI cohort over 4 years (3.5%) [1]. However, the percent of incident KOA attributable to AKOA may be slightly lower in the population-based cohort (15%) than the OAI (22%) [1]. It is unclear if the difference in the proportion of AKOA to incident KOA is attributable to Chingford participants being slightly younger or less obese than those in the OAI, other selection criteria, or the additional year of observation used to define AKOA in the Chingford Cohort (5 vs 4 years). Future endeavors that explore AKOA through cross-cohort collaborations may help explain the difference in proportion of AKOA between cohorts. Regardless, it is alarming that we consistently observe that at least 1 in 7 adults who develop KOA may experience an accelerated onset and progression of disease. The implications of this for clinical trials and epidemiological studies warrants further exploration.

Previously reported risk factors and outcomes related with AKOA in the OAI may be generalizable to a broader population. The current analyses supported prior findings that adults with AKOA are likely to have a greater age and BMI than adults with no KOA [1]. Furthermore, we’ve previously observed that age, and not BMI or blood pressure, was associated with AKOA when compared with typical KOA [1]. Within the OAI, we found a trend that blood pressure may be related to AKOA but post hoc analyses failed to support those findings [1]. Similarly, in the Chingford Cohort, we found no association between AKOA and blood pressure in our meta-analysis. Finally, we observed in Chingford and OAI that adults with AKOA may more frequently receive a knee replacement than their peers.

While the Chingford Cohort offered an excellent opportunity to explore AKOA, it is important to acknowledge several limitations. Firstly, the definition of AKOA was adapted to permit AKOA and typical KOA to develop over 5 years versus 4 years. However, we believed this was acceptable since 98% of people developed AKOA over 3 years in the OAI [3]. Secondly, the inter-observer agreement for radiographic severity was moderate to substantial. While the moderate agreement may increase the chance of misclassification, we believe this had minimal impact on our findings since our results complement prior results from the OAI. Thirdly, we could not determine the precise timing of AKOA and therefore it is unclear how much time elapsed between the onset of AKOA and total knee replacement. This limits our ability to compare the incidence of knee replacements after the onset of AKOA between Chingford and the OAI. We also could only explore 4 risk factors and one outcome in Chingford because we focused on variables that were consistently collected overtime in the OAI and Chingford. Despite this limitation, we showed considerable agreement in the findings between Chingford and OAI. The sample size also limited our ability to explore innovative questions about whether risk factors have different associations between those who develop bilateral or unilateral KOA. Future cross-cohort collaborations may provide a more nuanced understanding of risk factors and outcomes; such as the complex interactions among risk factors, which were observed in the OAI [8, 9] and may be inferred from Table 3.

## Conclusions

In conclusion, AKOA represents more than 1 in 7 women with incident KOA over 5 years. People with AKOA were more likely to have greater age and BMI prior to disease onset and were possibly more likely to receive a future knee replacement. These findings offer the first estimates of AKOA in a community-based cohort and confirm prior findings from the OAI. Considering the proportion of adults with incident KOA that may experience AKOA there is a critical need to understand how this subset of KOA influences findings from clinical trials and epidemiological studies.

## Supplementary information

**Additional file 1: Table S1.** Pooled Estimates (with Fixed and Random Effect) of Cumulative Incidence of Accelerated and Typical Knee Osteoarthritis (KOA) over 5-year intervals. **Table S2.** Meta-Analysis of Baseline Characteristics from the 3 Phases with Fixed- and Random-Effect. The supplemental tables offer the cumulative incidences and odds ratios from each period when pooled using fixed-effect or random-effect meta-analysis models to estimate the cumulative incidence and odds ratios.

## Data Availability

For information about the access to the Chingford 1000 Women Study data, please email chingford@ndorms.ox.ac.uk.

## Abbreviations

AKOA: Accelerated knee osteoarthritis
BMI: Body mass index
KL: Kellgren-Lawrence
KOA: Knee osteoarthritis
OAI: Osteoarthritis Initiative
OR: Odds ratio
