# Supplementary material for: The incidence and characteristics of accelerated knee osteoarthritis among women: the Chingford cohort
Source: BMC Musculoskelet Disord. 2020 Jan 31;21:60. doi: 10.1186/s12891-020-3073-3 (PMC6995080; doi:10.1186/s12891-020-3073-3)
Supplement: Supplementary file 1 — Additional file 1: Table S1. Pooled Estimates (with Fixed and Random Effect) of Cumulative Incidence of Accelerated and Typical Knee Osteoarthritis (KOA) over 5-year intervals. Table S2. Meta-Analysis of Baseline Characteristics from the 3 Phases with Fixed- and Random-Effect. The supplemental tables offer the cumulative incidences and odds ratios from each period when pooled using fixed-effect or random-effect meta-analysis models to estimate the cumulative incidence and odds ratios. [file 12891_2020_3073_MOESM1_ESM.docx]

| **Table S1. Pooled Estimates (with Fixed and Random Effect) of Cumulative Incidence of Accelerated and Typical Knee Osteoarthritis (KOA) over 5-year intervals** | | |
| --- | --- | --- |
|  | Accelerated  KOA | Typical  KOA |
| Person-based |  |  |
| **Fixed Effect (95% CI)** | 3.9% (3.0 to 4.9) | 21.7% (19.7 to 23.8) |
| **Random Effect (95% CI)** | 3.9% (2.9 to 5.3) | 20.7% (12.3 to 32.6) |
| Knee-based |  |  |
| **Fixed Effect (95% CI)** | 3.7% (3.1 to 4.3) | 17.6% (16.3 to 18.9) |
| **Random Effect (95% CI)** | 3.6% (2.2 to 5.7) | 16.4% (9.8 to 26.1) |

| **Table S2. Meta-Analysis of Baseline Characteristics from the 3 Phases with Fixed- and Random-Effect** | | | | |
| --- | --- | --- | --- | --- |
| Variable | AKOA vs No KOA | AKOA vs Typical KOA | AKOA vs No KOA | AKOA vs Typical KOA |
|  | OR (95% CI) | OR (95% CI) | aOR (95% CI) | aOR (95% CI) |
| **Fixed-Effect Meta-Analysis (From Table 3)** | | | | |
| Age (sd = 5.8 years) | **1.75 (1.35 to 2.26)** | **1.52 (1.16 to 2.01)** | **1.84 (1.40 to 2.43)** | **1.56 (1.16 to 2.11)** |
| BMI (sd = 3.9 kg/m^2^) | **1.47 (1.17 to 1.84)** | 1.11 (0.87 to 1.40) | **1.52 (1.17 to 1.97)** | 1.13 (0.86 to 1.48) |
| Systolic BP (sd = 20 mmHg) | 1.18 (0.93 to 1.50) | 1.18 (0.91 to 1.53) | 0.90 (0.63 to 1.28) | 1.06 (0.72 to 1.57) |
| Diastolic BP (sd = 11 mmHg) | 1.11 (0.87 to 1.43) | 1.01 (0.77 to 1.33) | 0.91 (0.63 to 1.31) | 0.79 (0.53 to 1.16) |
| **Random-Effect Meta-Analysis** | | | | |
| Age (sd = 5.8 years) | **1.75 (1.35 to 2.26)** | **1.53 (1.15 to 2.03)** | **1.89 (1.28 to 2.79)** | **1.59 (1.07 to 2.38)** |
| BMI (sd = 3.9 kg/m^2^) | **1.46 (1.07 to 1.98)** | 1.09 (0.75 to 1.58) | 1.56 (0.92 to 2.65) | 1.15 (0.60 to 2.19) |
| Systolic BP (sd = 20 mmHg) | 1.17 (0.90 to 1.54) | 1.15 (0.70 to 1.89) | 0.84 (0.45 to 1.54) | 0.99 (0.44 to 2.23) |
| Diastolic BP (sd = 11 mmHg) | 1.07 (0.73 to 1.58) | 0.97 (0.61 to 1.56) | 0.90 (0.39 to 2.08) | 0.81 (0.34 to 1.92) |
| Notes: AKOA = accelerated knee osteoarthritis, KOA = knee osteoarthritis, OR = odds ratio (per 1 standard deviation), 95% CI = 95% confidence interval, aOR = adjusted odds ratio (adjusted for other variables in table), sd = standard deviation, BMI = body mass index, BP = blood pressure | | | | |
